# First Report of Trametinib‐Nintedanib Combination in KRAS G12D‐Mutated Pancreatic Cancer: Efficacy and Fatal Hemorrhagic Complication: A Case Report

**DOI:** 10.1002/ccr3.71505

**Published:** 2025-11-26

**Authors:** Yufei Zhao, Li Yuan, Song Wang, Chensi Wu

**Affiliations:** ^1^ Department of Immunology and Rheumatology The Fourth Hospital of Hebei Medical University Shijiazhuang Hebei People's Republic of China; ^2^ Department of Endoscopy The Fourth Hospital of Hebei Medical University Shijiazhuang Hebei People's Republic of China; ^3^ Department of Gastroenterology The Fourth Hospital of Hebei Medical University Shijiazhuang Hebei People's Republic of China

**Keywords:** hemorrhagic toxicity, KRAS G12D mutation, MEK inhibitor, nintedanib, PDAC, trametinib

## Abstract

This is the first report of trametinib‐nintedanib for a 57‐year‐old KRAS G12D‐mutated recurrent pancreatic ductal adenocarcinoma. He had transient remission (lower CA19‐9, stable lesions) but died of gastrointestinal bleeding, showing efficacy and bleeding risk.

## 1 Introduction

Pancreatic ductal adenocarcinoma (PDAC) ranks among the most aggressive and lethal malignancies globally [1]. Pancreatic ductal adenocarcinoma (PDAC) is characterized by two key features in its tumor microenvironment: an abnormally abundant fibrotic stroma (also known as a “desmoplastic reaction”) and a highly immunosuppressive nature [2]. This dual microenvironmental profile frequently renders PDAC resistant to both targeted therapies and immunotherapies—a challenge compounded by the critical need for early diagnosis. Its subtle early symptoms often delay detection until the disease reaches advanced stages, at which point the five‐year survival rate drops below 5%, in contrast to over 20% for early‐stage PDAC [1, 3]. Kirsten ratsarcoma viral oncogene homolog (KRAS) mutation represents the most prevalent mutational subtype in human cancers; oncogenic KRAS mutations—particularly those affecting codons G12 and G13—impair GTPase activity, thereby driving constitutive pathway activation and aberrant signal transduction in cancer cells [2]. Approximately 90% of human PDAC cases harbor KRAS mutations: while targeted agents for KRAS G12C have now been approved for clinical use [4], inhibitors against other KRAS subtypes (e.g., G12D, G12X) remain in the clinical trial stage. The mitogen‐activated protein kinase (MAPK) pathway is one of the best‐characterized downstream signaling cascades of KRAS and has been extensively investigated as a therapeutic target for KRAS‐mutant tumors [5].

Nintedanib is a triple vascular kinase inhibitor that targets vascular endothelial growth factors (VEGFR)1/2/3, fibroblast growth factor receptor (FGFR)1/2/3, and platelet‐derived growth factor receptors (PDGFR)α/β signaling. In PDAC, nintedanib inhibits the proliferation of cells across multiple lineages, blocks PI3K/MAPK activity, and induces apoptosis, with these effects validated both in vitro and in vivo [6]. Previous studies, leveraging systematic high‐throughput combinatorial drug screening, identified a synergistic interaction between the mitogen‐activated protein kinase kinase (MEK) inhibitor trametinib and nintedanib—an interaction that specifically targets KRAS‐driven oncogenic signaling in mesenchymal PDAC [7]. This combination therapy thus holds promise as a novel therapeutic strategy for patients with the aggressive, refractory KRAS non‐G12C subtype of PDAC.

Herein, we report a case of a patient with postsurgical recurrent PDAC harboring the KRAS G12D mutation. Following the failure of multiple lines of prior treatment, the patient achieved a transient remission with trametinib in combination with nintedanib, but ultimately succumbed to gastrointestinal bleeding.

## 2 Case History/Examination

**FIGURE 1:**
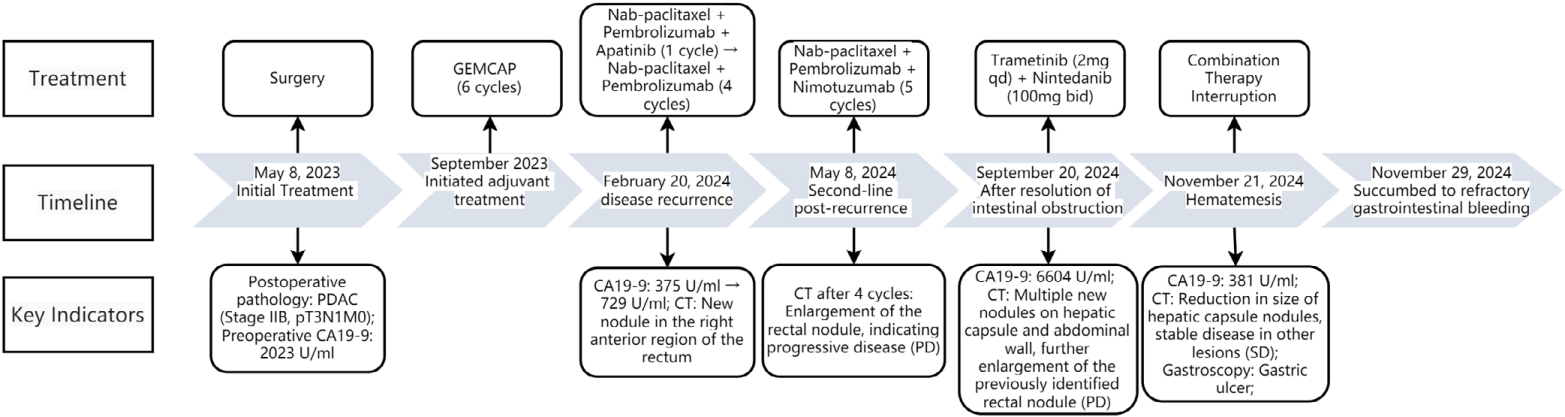
Treatment Timeline of the Patient.

**FIGURE 2:**
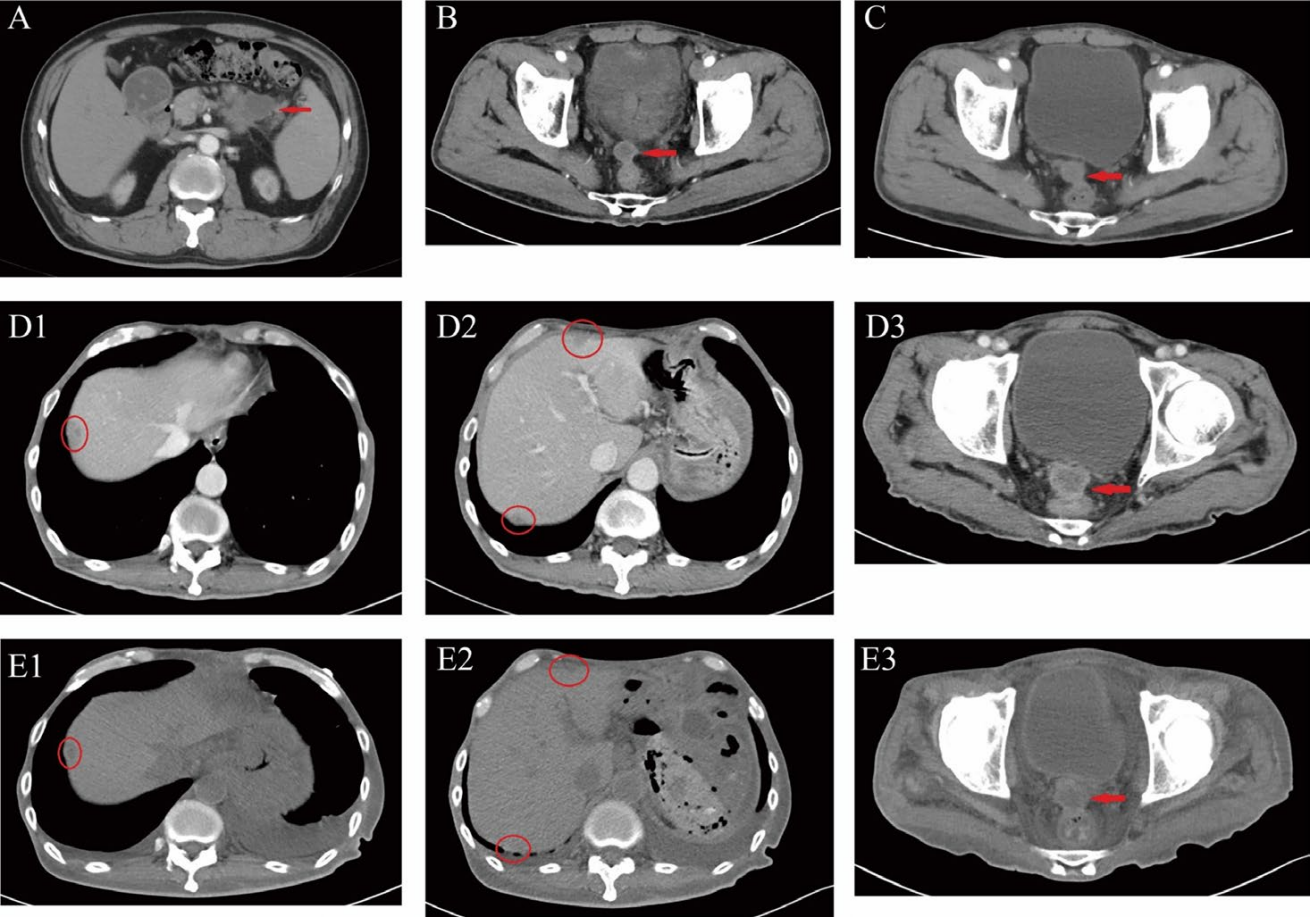
Serial imaging findings during treatment. A (2023‐04‐25, Baseline): Pancreatic tail mass (4.4 × 3.7 cm, arrow). B (2024‐02‐29, Post‐adjuvant therapy): New 1.8‐cm nodule anterior to the rectum (arrow). C (2024‐05‐07, Post‐first‐line chemotherapy): Rectal nodule progression (2.5 cm, arrow). D (2024‐09‐05, Post‐second‐line therapy): Multiple new nodules along the hepatic capsule and abdominal wall (circles, D1‐D2); rectal lesion further growth (2.9 cm, D3). E (2024‐11‐26, Post‐dual targeted therapy): Hepatic capsular nodules regression (circles, E1‐E2); abdominal wall and rectal lesions stable (rectal lesion: 2.8 cm, E3).

A 57‐year‐old Han Chinese male was admitted to hospital with complaints of abdominal distension. Laboratory tests revealed a markedly elevated carbohydrate antigen 19–9 (CA19‐9) level of 2023 U/mL (Figure 1). A routine gastroscopy is performed preoperatively to rule out gastric cancer and ulcers. Preoperative needle biopsy confirmed a diagnosis of pancreatic cancer (Figure 2A), and he underwent laparoscopic distal pancreatectomy on May 8, 2023. Postoperative pathological examination confirmed PDAC, with a pathological stage of IIB (pT3N1M0) according to the TNM classification system.

## 3 Differential Diagnosis, Investigations and Treatment

Postoperatively, the patient developed multiple complications, including intra‐abdominal hemorrhage, intra‐abdominal infection, bilateral pulmonary inflammation, and gastrointestinal bleeding. He was transferred to the Intensive Care Unit for supportive care and achieved complete recovery. Four months after surgery, he initiated adjuvant treatment with 6 cycles of the GEMCAP regimen (each cycle lasting 3 weeks), with the final cycle completed on January 20, 2024. On February 20, 2024, follow‐up assessment showed a significant increase in CA19‐9 from 375 U/mL to 729 U/mL (+94.4%). Abdominal computed tomography (CT) further revealed a new nodule in the right anterior region of the rectum (1.8 cm, Figure 2B), consistent with disease recurrence.

Subsequently, the treatment regimen was adjusted to nab‐paclitaxel, pembrolizumab, and apatinib (a small‐molecule anti‐angiogenic agent). However, apatinib was discontinued after 1 cycle due to febrile adverse events. Following 4 cycles of this modified regimen, re‐evaluation via abdominal CT demonstrated an increase in the size of the rectal nodule (2.5 cm, +38.9%, Figure 2C), indicating progressive disease. On May 8, 2024, the regimen was again revised to a 3‐week cycle of nab‐paclitaxel, pembrolizumab, and nimotuzumab, which was administered for 5 cycles; interim evaluation confirmed stable disease during this period.

On September 5, 2024, the patient was readmitted due to intestinal obstruction. Abdominal CT showed multiple new nodules on the hepatic capsule, multiple nodules on the abdominal wall, and further enlargement of the previously identified rectal nodule (2.9 cm, +16%, Figure 2D), confirming disease progression. His Eastern Cooperative Oncology Group (ECOG) score was 3, rendering him unsuitable for continued chemotherapy. At this time, his CA19‐9 level had risen to 6604 U/mL (+806.2%). Genetic testing was therefore performed, which detected a KRAS G12D mutation.

**FIGURE 3:**
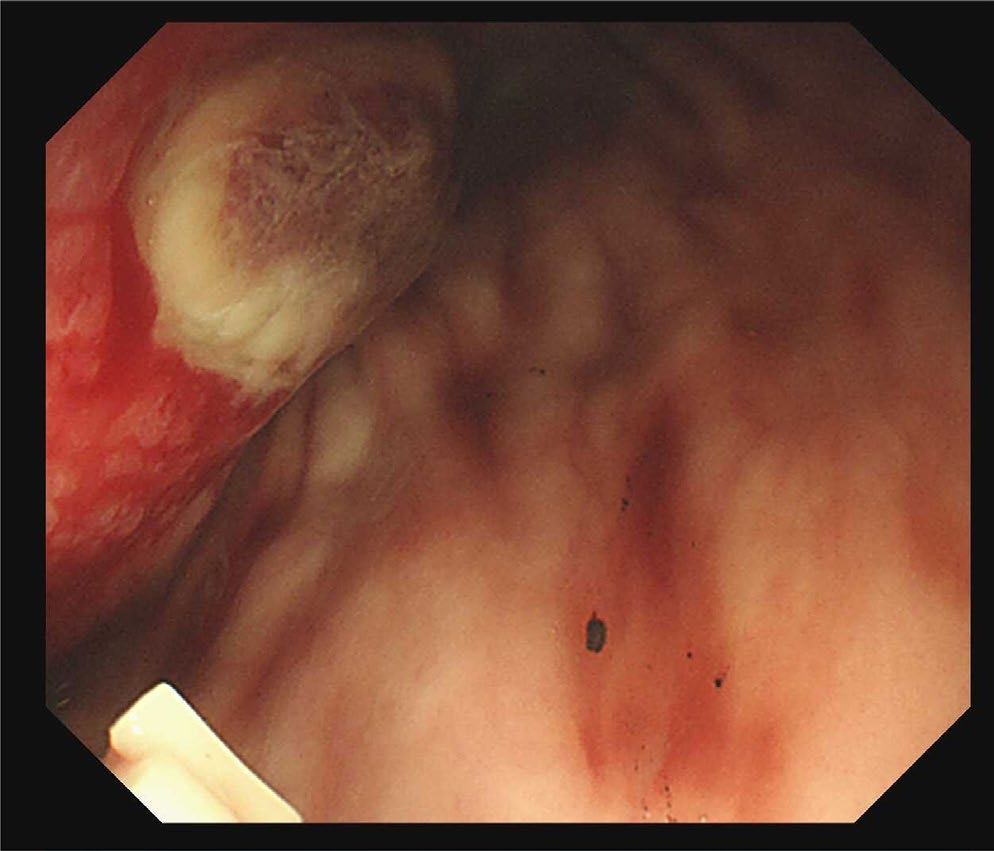
Gastroscopic manifestations following gastrointestinal hemorrhage.

After successful resolution of the intestinal obstruction and obtaining written informed consent from the patient's family, the patient was initiated on combination therapy with trametinib and nintedanib on September 20, 2024. First, the reason we chose the combination regimen of trametinib and nintedanib is based on the synergistic interaction identified between the two through systematic high‐throughput combinatorial drug screening. Additionally, we aim to modify the unique tumor microenvironment of pancreatic cancer and thereby overcome treatment resistance by leveraging the dual anti‐fibrotic and anti‐angiogenic effects of nintedanib. Before medication administration, we conducted complete blood count, liver function, and coagulation function tests, and the results showed no contraindications to medication use (Table 1). The dosage of our medication was determined based on the FDA‐recommended doses and the patient's physical status. Trametinib was administered at the standard dose of 2 mg once daily. However, for nintedanib, taking into account the patient's poor physical condition, low body weight, and FDA guidelines, the dose was reduced from the full FDA‐recommended dose (150 mg twice daily) to 100 mg twice daily. A follow‐up CT scan on October 28, 2024, showed stable lesions, accompanied by a significant improvement in the patient's physical status (ECOG score 2) and a marked decrease in CA19‐9 to 1779 U/mL (−73.1%). On November 21, 2024, the patient developed hematemesis. Given that the patient had not used anticoagulants or NSAIDs recently, we recommended discontinuing oral targeted drug therapy. At this point, CT imaging showed a reduction in the size of hepatic capsule nodules and stable disease in other lesions (2.8 cm, −3.4%, Figure 2E), while gastroscopy revealed a gastric ulcer (Figure 3). The gastroscopy revealed an inactive ulcer, so no endoscopic hemostasis was attempted. We implemented treatments including fasting, nutritional support, as well as medications for acid suppression, hemostasis, and splanchnic vasoconstriction. Additionally, after the bleeding episode, a total of 18 U of red blood cells were administered to correct anemia, and 10 U of cryoprecipitate to correct coagulation dysfunction. His CA19‐9 level had further decreased to 381 U/mL (−78.6%). Regrettably, despite aggressive hemostatic therapy and blood transfusion support, the patient succumbed to refractory gastrointestinal bleeding on November 29, 2024. This resulted in a total duration of trametinib–nintedanib combination therapy of approximately 2 months.

**TABLE 1:** Patient's Laboratory Monitoring Results During Treatment.

No.	Labs on admission (units)	Value				Reference range
		Before medication use	Routine monitoring	At the time of bleeding	One day before death	
		September 21st	October 26th	November 21st	November 28th	
1	white blood cell (x 109/L)	8.61	7.28	7.15	10.22	3.5–9.5
2	Hemoglobin (g/L)	109	96	63	78	130–175
3	Platelet (x 109/L)	365	244	146	134	125–350
4	Alanine Aminotransferase (U/L)	46.5	23.5	11.8	—	9–50
5	Aspartate Aminotransferase (U/L)	27.5	32.5	20.6	—	15–40
6	Albumin (g/L)	37.1	29.2	22.3	—	40–55
7	Total Bilirubin (umol/L)	4.6	6.9	4.5	—	0–26
8	Prothrombin time (seconds)	12.7	13.4	17.5	21	9.4–12.5
9	International normalized ratio	1.15	1.21	1.58	1.9	0.8–1.4

## 4 Conclusion and Results

This single case offers preliminary observational evidence that the trametinib‐nintedanib dual regimen may possess antitumor activity in a patient with KRAS G12D‐mutated PDAC, while also calling attention to the considerable bleeding risk linked to this combination. Owing to the inherent limitations of a single case (including the patient's fatal outcome), the findings reported here should be construed as hypothesis‐generating rather than conclusive evidence of the regimen's efficacy or safety. Additional controlled studies with larger patient cohorts are thus critical to verify whether this dual therapy might hold clinical potential for KRAS G12D‐mutated PDAC, and to better define and reduce its bleeding risk.

## 5 Discussion

PDAC is the most common subtype of pancreatic cancer, distinguished by highly intricate molecular characteristics. As a key proto‐oncogene, the KRAS gene plays an indispensable role in the initiation and progression of pancreatic cancer [8]. Specifically, the KRAS gene encodes a small‐molecule GTPase that acts as a central mediator in cellular signal transduction cascades. Under normal physiological conditions, KRAS protein is activated upon binding to guanosine triphosphate (GTP), thereby triggering downstream signaling pathways—most notably the RAS–RAF–MEK–ERK pathway—to regulate essential cellular processes such as proliferation, differentiation, and survival. In contrast, when GTP is hydrolyzed to guanosine diphosphate (GDP), the KRAS protein is rapidly inactivated, and subsequent signal transduction is terminated [9]. Mutations in the KRAS gene, however, disrupt this regulatory balance, leading to constitutive activation of the KRAS protein. This persistent activation drives aberrant cellular proliferation and ultimately contributes to tumorigenesis [2].

KRAS mutations are exceedingly prevalent in PDAC, with notable variations in subtype distribution and functional implications. Epidemiological data demonstrate that the majority of KRAS mutations in PDAC occur at codon 12, with the three most common variants being G12D (41%), G12V (32%), and G12R (16%) [10]. Despite their localization within the same codon, these mutations exhibit distinct biological effects and may correlate with differences in clinical prognosis. For instance, the G12D mutation has been shown to more potently activate the transcriptional program of the RAS signaling pathway compared to other codon 12 variants [10]. The constitutive activation of KRAS induced by such mutations amplifies downstream RAS–ERK signaling, fostering the uncontrolled proliferation of tumor cells and conferring resistance to conventional therapeutic modalities. Notably, direct targeting of KRAS remains challenging due to its unique structural properties—including the lack of accessible binding pockets—and its complex functional crosstalk with multiple signaling pathways [11]. In light of these obstacles, researchers have increasingly focused on strategies that target downstream signaling nodes or combine targeted agents to overcome therapeutic resistance.

MEK, the only direct substrate of rapidly‐accelerated fibrosarcoma (RAF) kinase, represents a critical regulatory node for inhibiting extracellular signal‐regulated kinase (ERK) signaling. Trametinib, a selective MEK inhibitor, exerts its therapeutic effect by specifically suppressing MEK kinase activity. However, monotherapy with trametinib has shown limited efficacy in RAS‐mutant tumors, primarily due to the emergence of feedback activation mechanisms—such as receptor tyrosine kinase (RTK)‐mediated reactivation of ERK signaling—that circumvent MEK inhibition [12]. Meanwhile, the fibrotic microenvironment is a well‐recognized hallmark of PDAC pathogenesis, contributing to tumor progression and treatment resistance [13]. The multi‐targeted kinase inhibitor nintedanib can block PDGFR, FGFR, and VEGFR signaling, reduce fibroblast proliferation and angiogenesis, thereby alleviating the fibrotic progression of PDAC [14].

Importantly, systematic high‐throughput combinatorial drug screening has identified a synergistic interaction between trametinib and nintedanib. This combination specifically targets KRAS‐driven oncogenic signaling in mesenchymal PDAC subtypes, induces tumor cell death, and mediates extensive reprogramming of the immunosuppressive tumor microenvironment—key mechanisms that enhance therapeutic efficacy [7]. Consistent with these preclinical findings, our KRAS G12D‐mutant patient (a subtype associated with robust RAS pathway activation) exhibited clinical remission signals, confirming the regimen's ability to target oncogenic KRAS signaling in heavily pretreated mesenchymal‐like PDAC. Notably, Falcomatà et al. demonstrated that this combination reduces fibrotic stroma via nintedanib's anti‐angiogenic/anti‐fibrotic effects and overcomes trametinib resistance by blocking feedback RTK activation, which likely underlies our patient's response despite prior treatment failure [7].

From the perspective of pharmacodynamic principles, the synergistic effect of the two essentially stems from complementary crosstalk at the mechanistic level. On the one hand, it is the dual targeting of signaling pathways and the microenvironment: trametinib blocks the RAS–RAF–MEK–ERK pathway from the “inside of cells” to inhibit the autonomous proliferation of tumors, while nintedanib blocks VEGFR signaling from the “external microenvironment” to cut off nutrient supply. At the same time, it improves the fibrotic matrix by inhibiting PDGFR and FGFR, creating conditions for trametinib to penetrate tumor tissues and reach target cells [7, 14]. On the other hand, it is the crosstalk regulation and compensatory inhibition between pathways: preclinical studies have shown that the use of anti‐VEGFR drugs alone can upregulate MEK–ERK signaling through a compensatory mechanism (such as activating the RTK bypass pathway) to maintain tumor survival [15], while the use of anti‐MEK drugs alone may increase VEGF secretion by feedback activation of hypoxia‐inducible factor (HIF‐1α) to promote angiogenesis [16]. However, the combination of the two can block this compensatory activation between pathways, prevent the activation of alternative survival pathways, and form a “cross‐inhibition” effect, which is also the key reason why the efficacy of combination therapy is superior to that of single‐agent therapy in KRAS‐driven models [7].

The combination of anti‐MEK and anti‐VEGFR agents blocks adaptive resistance by targeting the core resistance pathways of monotherapy: During anti‐MEK monotherapy, tumors often upregulate the expression or activity of RTKs such as EGFR, FGFR, and PDGFR to reactivate ERK signaling and bypass MEK inhibition—this phenomenon is more pronounced in mesenchymal PDAC due to higher baseline RTK expression [7, 12]. Nintedanib reverses this resistance through dual mechanisms: it directly inhibits FGFR and PDGFR activity, and reduces the availability of RTK ligands (e.g., FGF, PDGF) in the tumor microenvironment (TME) via its anti‐angiogenic effect. This is critical for the KRAS G12D‐mutant patient in this study, as this mutation enhances RTK signal crosstalk [10]; without this combination, trametinib monotherapy would likely fail due to resistance. For anti‐VEGFR monotherapy, tumors tend to develop adaptive vascular remodeling (e.g., switching to FGF dependence) and increased stromal fibrosis (driven by PDGFR‐activated fibroblasts) to re‐establish blood supply, leading to resistance [17]. Trametinib addresses this by inhibiting the MEK–ERK pathway to reduce FGF secretion by tumor cells and suppress stromal fibroblast proliferation, disrupting the compensatory cycle. Preclinical models have confirmed that the combination of these two agents achieves sustained tumor vascular normalization [7].

The mechanism of gastrointestinal bleeding caused by the combination of MEK inhibitors and vascular kinase inhibitors is a triple superimposition of “drug target effect + tumor microenvironment abnormality + insufficient individual patient reserve”: Nintedanib disrupts vascular structure and repair by inhibiting VEGFR/PDGFR [18]; trametinib impairs the proliferation and repair capacity of mucosal cells by suppressing the MEK–ERK pathway [19]; and mucosal damage, cachexia, and a history of multiple lines of treatment associated with advanced tumors further lower the patient's bleeding threshold, ultimately inducing refractory gastrointestinal bleeding.

To the best of our knowledge, this case is the first globally reported instance of a KRAS G12D‐mutant PDAC patient treated with the combination of trametinib and nintedanib. The patient's condition was stable and showed a trend toward remission, as evidenced by improvements in clinical symptoms, radiological stabilization/reduction of lesions, and a significant decline in the tumor marker CA19‐9. These findings provide valuable real‐world evidence supporting the tumor‐suppressive potential of this combination therapy for KRAS G12D‐mutant PDAC—a subtype for which effective targeted treatments remain scarce. Regrettably, the patient ultimately died from gastrointestinal bleeding. While it is difficult to definitively attribute this complication solely to the therapy (given the patient's advanced‐stage disease and potential cachectic state), the observed bleeding risk raises concerns that may limit the widespread clinical adoption of this combination regimen.

This case underscores the promising role of trametinib‐nintedanib combination therapy as a targeted approach for KRAS G12D‐mutant PDAC, addressing an unmet clinical need. However, critical gaps remain in understanding its safety profile—particularly the incidence and mechanisms of gastrointestinal bleeding. Future research should prioritize several key areas: (1) evaluating this combination in larger patient cohorts to better characterize its efficacy and safety; (2) optimizing dosage regimens to balance therapeutic benefit and toxicity; (3) identifying predictive biomarkers that can distinguish patients likely to respond to treatment or develop adverse events; and (4) exploring combinatorial strategies with anti‐bleeding agents or supportive care interventions to mitigate bleeding risk. Addressing these areas will be essential to translating the therapeutic potential of this regimen into improved clinical outcomes for patients with KRAS non‐G12C‐mutant PDAC.

While this case documents notable antitumor signals (e.g., CA19‐9 reduction, radiological lesion stabilization) and a concerning bleeding event with trametinib‐nintedanib, these observations have key limitations constraining their interpretability and generalizability. First, as a single‐patient report, findings cannot be generalized to broader KRAS G12D‐mutated PDAC populations—individual variability in tumor biology, co‐morbidities, and prior treatments may uniquely drive responses or adverse events. Second, the lack of post‐treatment biopsy confirmation limits mechanistic insight: while radiological/tumor marker changes suggest activity, pathological validation (e.g., proliferation, apoptosis) was absent. Combined with unobtained metastatic lesion pathology (due to tolerance/financial constraints), this weakens molecular‐clinical correlations. Third, short therapy duration hinders assessment of response durability and late‐onset adverse events—we cannot confirm if stabilization would persist or if additional risks would emerge long‐term. Fourth, no pharmacogenomic analyses mean we cannot link interindividual differences (e.g., drug metabolism, target genetics) to efficacy/toxicity, a critical gap for personalized strategies.

Given these limitations, this report's primary value is generating testable hypotheses (viability of MEK/angiokinase dual inhibition for KRAS G12D‐mutated PDAC, bleeding risk management) rather than proving clinical utility. Future studies should address these via larger cohorts, correlative pathological/pharmacogenomic analyses, and longer follow‐up to characterize benefit–risk profiles.

## Author Contributions

**Yufei Zhao:** project administration, writing – original draft. **Li Yuan:** data curation, investigation. **Song Wang:** data curation. **Chensi Wu:** conceptualization, writing – review and editing.

## Funding

The authors received no specific funding for this work.

## Consent

Written informed consent has been obtained.

## Conflicts of Interest

The authors declare no conflicts of interest.

## Data Availability

The datasets used during the current study are available from the corresponding author upon reasonable request.

## References

[1] Y. Lin , S. Pu , J. Wang , et al., “Pancreatic STAT5 Activation Promotes Kras(G12D)‐Induced and Inflammation‐Induced Acinar‐To‐Ductal Metaplasia and Pancreatic Cancer,” Gut 73 (2024): 1831–1843. 38955401 10.1136/gutjnl-2024-332225 PMC11503187

[2] K. Mondal , M. K. Posa , R. P. Shenoy , and S. Roychoudhury , “KRAS Mutation Subtypes and Their Association With Other Driver Mutations in Oncogenic Pathways,” Cells 13 (2024): 1221. 39056802 10.3390/cells13141221 PMC11274496

[3] A. Deipenbrock , B. E. Wilmes , T. Sommermann , et al., “Modelling of the Multicellular Tumor Microenvironment of Pancreatic Ductal Adenocarcinoma (PDAC) on a Fit‐For‐Purpose Biochip for Preclinical Drug Discovery,” Lab on a Chip 25 (2025): 2168–2181. 40018951 10.1039/d4lc01016g

[4] J. H. Strickler , H. Satake , T. J. George , et al., “Sotorasib in KRAS p.G12C‐Mutated Advanced Pancreatic Cancer,” New England Journal of Medicine 388 (2023): 33–43. 36546651 10.1056/NEJMoa2208470 PMC10506456

[5] M. Drosten and M. Barbacid , “Targeting the MAPK Pathway in KRAS‐Driven Tumors,” Cancer Cell 37 (2020): 543–550. 32289276 10.1016/j.ccell.2020.03.013

[6] N. Awasthi , S. Hinz , R. A. Brekken , M. A. Schwarz , and R. E. Schwarz , “Nintedanib, a Triple Angiokinase Inhibitor, Enhances Cytotoxic Therapy Response in Pancreatic Cancer,” Cancer Letters 358 (2015): 59–66. 25527450 10.1016/j.canlet.2014.12.027 PMC4450873

[7] C. Falcomatà , S. Bärthel , S. A. Widholz , et al., “Selective Multi‐Kinase Inhibition Sensitizes Mesenchymal Pancreatic Cancer to Immune Checkpoint Blockade by Remodeling the Tumor Microenvironment,” Nature Cancer 3 (2022): 318–336. 35122074 10.1038/s43018-021-00326-1 PMC7612546

[8] A. M. Varghese , M. A. Perry , J. F. Chou , et al., “Clinicogenomic Landscape of Pancreatic Adenocarcinoma Identifies KRAS Mutant Dosage as Prognostic of Overall Survival,” Nature Medicine 31 (2025): 466–477. 10.1038/s41591-024-03362-3 PMC11835752 39753968

[9] H. J. Kim , H. N. Lee , M. S. Jeong , and S. B. Jang , “Oncogenic KRAS: Signaling and Drug Resistance,” Cancers 13 (2021): 5599. 34830757 10.3390/cancers13225599 PMC8616169

[10] J. K. Lennerz and A. Stenzinger , “Allelic Ratio of KRAS Mutations in Pancreatic Cancer,” Oncologist 20 (2015): e8–e9. 25777349 10.1634/theoncologist.2014-0408 PMC4391769

[11] A. Takashima and D. V. Faller , “Targeting the RAS Oncogene,” Expert Opinion on Therapeutic Targets 17 (2013): 507–531. 23360111 10.1517/14728222.2013.764990 PMC3804031

[12] U. Degirmenci , M. Wang , and J. Hu , “Targeting Aberrant RAS/RAF/MEK/ERK Signaling for Cancer Therapy,” Cells 9 (2020): 198. 31941155 10.3390/cells9010198 PMC7017232

[13] Y. W. Tien , Y. M. Wu , W. C. Lin , H. S. Lee , and P. H. Lee , “Pancreatic Carcinoma Cells Stimulate Proliferation and Matrix Synthesis of Hepatic Stellate Cells,” Journal of Hepatology 51 (2009): 307–314. 19464749 10.1016/j.jhep.2009.03.016

[14] S. Wind , U. Schmid , M. Freiwald , et al., “Clinical Pharmacokinetics and Pharmacodynamics of Nintedanib,” Clinical Pharmacokinetics 58 (2019): 1131–1147. 31016670 10.1007/s40262-019-00766-0 PMC6719436

[15] L. B. Zhou , Y. Q. Zhou , and X. Y. Zhang , “Blocking VEGF Signaling Augments Interleukin‐8 Secretion via MEK/ERK/1/2 Axis in Human Retinal Pigment Epithelial Cells,” International Journal of Ophthalmology 13 (2020): 1039–1045. 32685389 10.18240/ijo.2020.07.04 PMC7321944

[16] X. M. Yang , Y. S. Wang , J. Zhang , et al., “Role of PI3K/Akt and MEK/ERK in Mediating Hypoxia‐Induced Expression of HIF‐1α and VEGF in Laser‐Induced Rat Choroidal Neovascularization,” Investigative Ophthalmology & Visual Science 50 (2009): 1873–1879. 19098317 10.1167/iovs.08-2591

[17] Y. Zhao and A. A. Adjei , “Targeting Angiogenesis in Cancer Therapy: Moving Beyond Vascular Endothelial Growth Factor,” Oncologist 20 (2015): 660–673. 26001391 10.1634/theoncologist.2014-0465 PMC4571783

[18] G. Grześk , A. Woźniak‐Wiśniewska , J. Błażejewski , et al., “The Interactions of Nintedanib and Oral Anticoagulants‐Molecular Mechanisms and Clinical Implications,” International Journal of Molecular Sciences 22 (2020): 282. 33396592 10.3390/ijms22010282 PMC7795697

[19] K. Gullapalli , O. Mosalem , M. T. Varghese , K. Watat , and B. Hrinczenko , “Severe Epistaxis Secondary to Dabrafenib and Trametinib Toxicity in Non‐Small Cell Lung Carcinoma With Small Bowel Metastasis,” Cureus 13 (2021): e16431. 34466299 10.7759/cureus.16431 PMC8396418

